# Relationship between food triggers and sensory hypersensitivity in patients with migraine

**DOI:** 10.1055/s-0044-1793934

**Published:** 2024-12-10

**Authors:** Aline Vitali-Silva, Valéria Aparecida Bello, Regina Célia Poli-Frederico, Carlos Eduardo Coral de Oliveira, Edna Maria Vissoci Reiche, Beatriz Bagatim Bossa, Debora Villas Boas Rezende, Bárbara Ferreira Khouri, Raimundo Pereira Silva-Néto

**Affiliations:** 1 Pontifícia Universidade Católica do Paraná, Escola de Medicina e Ciências da Vida, Curso de Medicina, Londrina PR, Brazil.; 2 Hospital Evangélico de Londrina, Londrina PR, Brazil.; 3 Universidade Federal do Delta do Parnaíba, Curso de Medicina, Departamento de Neurologia, Parnaíba PI, Brazil.

**Keywords:** Migraine Disorders, Photophobia, Hyperacusis, Olfaction Disorders, Precipitating Factors, Food, Sensation Disorders, Transtornos de Enxaqueca, Fotofobia, Hiperacusia, Transtornos do Olfato, Fatores Desencadeantes, Alimentos, Transtornos de Sensação

## Abstract

**Background** The recognition of food as the trigger of attacks occurs in approximately 25% of individuals with migraine. However, differentiating migraine food triggers and prodrome symptoms is still a challenge.

**Objective** To understand the association of clinical characteristics of migraine with food triggers and to identify predictors of food triggers.

**Methods** Patients with migraine diagnosed according to the criteria of the third edition of the International Classification of Headache Disorders (ICHD-3) were evaluated for the presence or absence of food triggers.

**Results** In total, 502 patients with migraine were investigated, and they were divided into two groups: those with food triggers (58.4%) and those without food triggers (41.6%). The main food triggers were alcohol (44%), chocolate (42%), cheese (27.7%), excess carbohydrates (27.7%), coffee (21.8%), cold cuts (16%), and citrus fruits (11.9%). Aura and excessive use of analgesics were more frequent among patients with food triggers ( *p* = 0.022). Photophobia and osmophobia were associated with the presence of a food trigger ( *p* < 0.001). There was a greater impact of migraine in the presence of food triggers ( *p* = 0.002). Through binary logistic regression, we identified clinical predictors of food triggers, such as photophobia and osmophobia.

**Conclusion** The presence of a food trigger was significantly associated with photophobia and osmophobia. Osmophobia might be another mechanism by which patients perceive foods as triggers for their migraine attacks.

## Resumo

**Antecedentes** A percepção dos gatilhos alimentares ocorre em aproximadamente 25% dos indivíduos com migrânea. No entanto, diferenciar os desencadeantes alimentares da migrânea e os sintomas prodrômicos ainda é um desafio.

**Objetivo** Compreender a associação de características clínicas da migrânea com gatilhos alimentares, bem como identificar preditores destes gatilhos.

**Métodos** Pacientes com migrânea diagnosticados de acordo com os critérios da terceira edição da Classificação Internacional de Cefaleias (International Classification of Headache Disorders, third edition, ICHD-3, em inglês) foram avaliados quanto à presença ou ausência de gatilhos alimentares.

**Resultados** Foram investigados 502 pacientes com migrânea, que foram divididos em 2 grupos: aqueles com gatilhos alimentares (58,4%) e aqueles sem gatilhos alimentares (41,6%). Os principais desencadeantes alimentares foram álcool (44%), chocolate (42%), queijo (27,7%), excesso de carboidratos (27,7%), café (21,8%), embutidos (16%) e frutas cítricas (11,9%). Aura e uso excessivo de analgésicos foram mais frequentes entre pacientes com gatilhos alimentares ( *p* = 0,022). Fotofobia e osmofobia estiveram associadas à presença de gatilho alimentar ( *p* < 0,001). Houve maior impacto da migrânea na presença de gatilhos alimentares ( *p* = 0,002). Por meio de regressão logística binária, foram identificados preditores clínicos de gatilhos alimentares, como fotofobia e osmofobia.

**Conclusão** A presença de um gatilho alimentar foi significativamente associada à fotofobia e à osmofobia. A osmofobia poderia ser outro mecanismo pelo qual os pacientes percebem os alimentos como gatilhos para suas crises de migrânea.

## INTRODUCTION

Migraine is a genetically-determined neurological disorder that predisposes individuals to recurrent episodes of typically intense, pulsating, unilateral headache accompanied by phonophobia, photophobia, and nausea. 1

Various behavioral, nutritional, and emotional factors are perceived as triggers for migraine attacks; however, there is limited compelling data to support these associations. 2 On the other hand, neurofunctional imaging studies 3 have demonstrated brain alterations that could lead to behavioral, dietary, and mood changes in the prodromal phase, which precedes migraine headache attacks. Thus, differentiating migraine triggers from prodromal symptoms remains a major challenge in the neurological practice.

It is a fact that the perception of dietary triggers occurs in approximately 25% of individuals with migraine. 2 There are several theories to explain this perception:

- there is a craving associated with the prodrome, wherein brain changes lead individuals to overconsume certain types of foods, especially sweets, with the ingestion of these foods being caused by the migraine itself rather than being a true trigger; 4
- there is an increase in nitric oxide availability, for example, during watermelon consumption, through activation of the L-arginine-nitric oxide pathway; 5
- there is an allergic mechanism mediated by immunoglobulins G (IgG) or E (IgE) against certain foods; 6 and
- the composition of oral and intestinal flora with nitrate, nitrite, and nitric oxide-reducing bacteria could increase the likelihood of migraine. 7

Despite the widely-acknowledged association between food and migraine, there is scant literature on this topic, and, to date, there are no studies clinically characterizing individuals who perceive dietary triggers. The aim of the present study was to explore the clinical characteristics of migraine patients associated with food triggers, as well as to identify predictors of dietary triggers.

## METHODS

### Study design and patients

The present was a prospective cross-sectional study with a group comparison. The study population comprised a non-random, convenience sampling of the first 524 migraine patients diagnosed by a neurologist according to the criteria of the third edition of the International Classification of Headache Disorders (ICHD-3). 1 They were cared for at the Headache Outpatient Clinic of the Pontifícia Universidade Católica do Paraná (PUCPR), in the city of Londrina, Brazil, and invited to participate in this research from December 2018 to June 2023.

### Inclusion and exclusion criteria

Patients aged 18 to 69 years with episodic or chronic migraine, with or without aura, were included according to the diagnostic criteria of the ICHD-3. 1 Participants with severe and uncontrolled systemic and/or psychiatric diseases, as well as pregnant women, were excluded from the study.

### Data collection

The patients provided a comprehensive medical history to a headache specialist, who recorded clinical, personal, and anthropometric data. Headache attacks were characterized according to the diagnostic criteria of the ICHD-3, 1 obtaining information on the presence of aura, nausea, phonophobia, photophobia, osmophobia, and presence of dietary triggers. After excluding the subjects with incomplete clinical data for the present study (22 patients), only 502 patients comprised the final sample. They were divided into two groups, according to the presence or absence of dietary triggers.

We considered that the patient was on preventive treatment when continuously using, for at least 30 consecutive days, topiramate, valproate, divalproex, amitriptyline, nortriptyline, venlafaxine, propranolol, atenolol, metoprolol, or flunarizine. Medication overuse was considered when the patient ingested a triptan, opioid, dihydroergotamine, or combination analgesic for ≥ 10 days per month, or non-opioid analgesics or nonsteroidal anti-inflammatory drugs for ≥ 15 days per month, according to ICHD-3 criteria. 1

The patients also filled out self-administered validated questionnaires to assess migraine-related disability through the Migraine Disability Assessment (MIDAS) 8 and migraine impact through the 6-Item Headache Impact Test (HIT-6). 9 The presence of allodynia was measured using the 12-Item Allodynia Symptom Checklist (ASC-12), 10 anxiety symptoms were assessed using the State-Trait Anxiety Inventory (STAI 1 and 2), 11 and depression was measured using the Beck Depression Inventory (BDI). 12 Finally, the participants completed the hyperacusis scale. 13

### Food triggers

The participants were asked about their perception of whether any food triggered a migraine attack. The interviewer read a list of foods, and the patient mentioned the food they considered triggering. This list included the following foods: cheese, chocolate, citrus fruits, red wine, white wine, beer, spirits, coffee, cold cuts, salami, monosodium glutamate, milk, dairy products, soft drinks, ice cream, nuts, chestnuts, almonds, sweetener, tomato, excessive carbohydrates, and fermented products (yogurts and similar foods). Excess carbohydrates were considered when the patient mentioned consuming foods high in carbohydrates, such as sweets and pasta, in greater quantities than usual. Participants were also given the option to add other foods not mentioned in the list.

### Ethical aspects

The present study was approved by the Ethics in Research Involving Human Subjects Committee at PUCPR, under protocol number 4.293.039, and the Presentation Certificate to Ethics Assessment, under registry number 98316718.7.0000.0020. All participants signed the informed consent form.

### Statistical analysis

Once the information was organized in the database, we used the IBM SPSS Statistics for Windows (IBM Corp., Armonk, NY, United States) software, version 28.0, for the statistical analysis. The categorical data were expressed as absolute numbers and percentages, and the continuous data, as mean and standard deviation values. The Chi-squared test with Yates correction, the Fisher's exact test and the Mann–Whitney test for differences between averages of unpaired samples were used, assuming a significance level of 0.05. The Student's *t* -test was used to analyze the clinical assessment scales. Binary logistic regression (enter method) was performed to identify variables associated with the perception of food triggers in two models: model 1 included demographic characteristics combined with clinical features of migraine that had a *p* -value < 0.1 in the univariate analyses; the second model included only these clinical features.

## RESULTS

Of the 502 migraine patients, 58.4% (293/502) reported that foods were triggers for headache attacks, while 41.6% (209/502) did not have this trigger. There were no statistical differences in terms of age, sex, ethnicity, and body mass index between patients with and without food triggers. The sample data are shown in Table 1 .

**Table 1:** Sociodemographic characteristics of 502 patients with migraine according to the presence or absence of food triggers

Characteristics		Comparison groups		*p* -value
		With food trigger (n = 293)	Without food trigger (n = 209)	
Sex: n (%)	Male	38 (13.0)	33 (15.8)	0.371
	Female	255 (87.0)	176 (84.2)	
Age (years)	Mean(± SD)	35(± 13)	34(± 12)	0.413
	Range	18–65	18–69	
Ethnicity: n (%)	Caucasian: n (%)	230 (78.5)	154 (73.7)	0.210
	Non-Caucasian: n (%)	63 (21.5)	55 (26.3)	
Body mass index: n (%)	Underweight	16 (5.5)	7 (3.3)	0.748
	Healthy weight	128 (43.7)	93 (44.5)	
	Overweight	91 (31.0)	61 (29.2)	
	Obesity	51 (17.4)	42 (20.1)	
	No information	7 (2.4)	6 (2.9)	

Abbreviation: SD, standard deviation.

Note: *p* -values based on the Chi-squared and Mann–Whitney tests.

Alcohol was the most frequently reported food trigger (44%; 129/293), followed by chocolate (42%; 123/293), cheese (27.7%; 81/293), excessive carbohydrates (27.7%; 81/293), coffee (21.8%; 64/293), cold cuts (16.0%; 47/293), citrus fruits (11.9%; 35/293), monosodium glutamate (7.5%; 22/293), and sweeteners (7.2%; 21/293) ( Table 2 ).

**Table 2:** Distribution of food triggers in 293 patients with migraine

Food triggers	Frequency	
	n	%
Alcohol	129	44.0
Chocolate	123	42.0
Cheese	81	27.7
Excess carbohydrates	81	27.7
Coffee	64	21.8
Cold cuts	47	16.0
Citrus fruits	35	11.9
Monosodium glutamate	22	7.5
Sweetener	21	7.2
Other foods	174	59.4

The clinical characteristics of migraine are presented in Table 3 . Aura and prophylactic medication use were more frequent among patients with food triggers compared to those without food triggers respectively: 137/293 (46.8%) versus 67/209 (32.1%) ( *p* = 0.001) and 113/293 (38.6%) versus 60/209 (28.7%) ( *p* = 0.022). There was no statistical difference in the prevalence of chronic migraine or medication overuse between the groups. The age at migraine onset was lower (19 ± 10 years versus 21 ± 11 years; *p* = 0.006) and the duration of illness was longer (18 ± 14 years versus 13 ± 11 years; *p* = 0.001) among individuals with food triggers compared to those without food triggers, respectively. There was no difference in the number of headache days or days of disabling pain. Photophobia and osmophobia were higher among individuals with food triggers compared to those without food triggers respectively: 278/293 (94.9%) versus 184/209 (88%) ( *p* = 0.005) and 228/293 (77.8%) versus 117/209 (56%) ( *p* < 0.001).

**Table 3:** Clinical characteristics of migraine in patients with and without food triggers

Parameters		Comparison groups		*p* -value
		With food trigger (n = 293)	Without food trigger (n = 209)	
Classification: n (%)	Episodic	155 (52.9)	118 (56.5)	0.430
	Chronic	138 (47.1)	91 (43.5)	
Aura: n (%)	No	156 (53.2)	142 (67.9)	0.001
	Yes	137 (46.8)	67 (32.1)	
Prophylactic medication: n (%)	No	180 (61.4)	149 (71.3)	0.022
	Yes	113 (38.6)	60 (28.7)	
Medication overuse: n (%)	No	161 (54.9)	124 (59.3)	0.475
	Yes	115 (39.3)	71 (34.0)	
	No information	17 (5.8)	14 (6.7)	
Frequency of headaches (days)	Mean(± SD)	11(± 9)	11(± 10)	0.323
	Range	0–31	0–39	
Frequency of disability headache (days)	Mean(± SD)	4(± 5)	4(± 5)	0.389
	Range	0–30	0–30	
Age at onset of migraine (years)	Mean(± SD)	19(± 10)	21(± 11)	0.006
	Range	3–60	4–61	
Duration of migraine (years)	Mean(± SD)	18(± 14)	13(± 11)	0.001
	Range	0–58	0–57	
Phonophobia: n (%)	No	35 (11.9)	37 (17.7)	0.070
	Yes	258 (88.1)	172 (82.3)	
Photophobia: n (%)	No	15 (5.1)	25 (12.0)	0.005
	Yes	278 (94.9)	184 (88.0)	
Osmophobia: n (%)	No	65 (22.2)	92 (44.0)	< 0.001
	Yes	228 (77.8)	117 (56.0)	

Abbreviation: SD, standard deviation.

Note: *p* -values based on the Chi-squared and Mann–Whitney tests.

Patients with food triggers had a higher mean migraine impact score (64 ± 6) compared to those without food triggers (62 ± 9) ( *p* = 0.002). There were no differences in terms of anxiety, depression, disability, allodynia, or hyperacusis scores ( *p* > 0.05) ( Table 4 ).

**Table 4:** Analysis of scales validated in individuals with migraine according to the presence of a food trigger

Questionnaires and scores	Comparison groups		*p* -value
	With food trigger (n = 293)	Without food trigger (n = 209)	
State-Trait Anxiety Inventory (STAI 1): mean ± SD	45(± 10)	44(± 12)	0.618
Range	24–69	20–80	
State-Trait Anxiety Inventory (STAI 2): mean ± SD	45(± 11)	45(± 11)	0.696
Range	22–98	22–79	
Beck Depression Inventory (BDI): mean ± SD	13(± 11)	12(± 9)	0.881
Range	0–130	0–47	
Migraine Disability Assessment (MIDAS): mean ± SD	34(± 40)	29(± 40)	0.175
Range	0–330	0–314	
6-Item Headache Impact Test (HIT 6): mean ± SD	64(± 6)	62(± 9)	0.002
Range	47–76	5–78	
12-Item Allodynia Symptom Checklist (ASC-12): mean ± SD	6(± 4)	6(± 4)	0.08
Range	0–18	0–22	
Hyperacusis Scale: mean ± SD	20(± 10)	19(± 11)	0.140
Range	0–42	0–42	

Abbreviation: SD, standard deviation.

Note: *p* -value based on the Student's *t* -test.

The binary logistic regression aimed at identifying clinical predictors of food triggers is presented in 2 models in Table 5 . Increasing age was associated with protection, while longer migraine duration, presence of photophobia and osmophobia, and higher migraine impact were associated with increased odds of food triggers. Notably, the presence of photophobia was associated with 3-fold higher odds of presenting food triggers (odds ratio [OR] = 3.31; 95% confidence interval [95%CI] = 1.26–8.66; *p* = 0.015 in model 1, and OR = 3.22; 95%CI = 1.26–8.18; *p* = 0.014 in model 2), and osmophobia was associated with 2-fold higher odds of presenting food triggers (OR = 2.64; 95%CI = 1.58–4.41; *p* < 0.001 in model 1, and OR = 2.45; 95%CI = 1.50–4.02; *p* < 0.001 in model 2).

**Table 5:** Analysis of food trigger predictors using binary logistic regression

Models		B	SE	Wald	df	OR (95%CI)	*p* -value
**Model 1**							
Age		−0.085	0.042	4.048	1	0.919 (0.846–0.998)	0.044
Sex		−0.045	0.355	0.016	1	0.956 (0.476–1.918)	0.899
Ethnicity		−0.528	0.290	3.312	1	0.590 (0.334–1.041)	0.069
BMI	Overweight	0.124	0.270	0.211	1	1.132 (0.667-1.919)	0.646
	Obesity	0.113	0.327	0.120	1	1.120 (0.590-2.123)	0.730
	Underweight	0.695	0.569	1.496	1	2.005 (0.658-6.110)	0.221
Aura		0.296	0.240	1.517	1	1.344 (0.839–2.154)	0.218
Prophylactic Medication		0.261	0.253	1.067	1	1.299 (0.791–2.132)	0.302
Age at onset of migraine		0.068	0.043	2.503	1	1.070 (0.984–1.164)	0.114
Duration of migraine		0.097	0.040	5.755	1	1.102 (1.018–1.193)	0.016
Photophobia		1.196	0.492	5.916	1	3.306 (1.261–8.663)	0.015
Phonophobia		−0.082	0.337	0.059	1	0.921 (0.475–1.784)	0.808
Osmophobia		0.970	0.262	13.700	1	2.638 (1.578–4.410)	< 0.001
HIT-6		0.035	0.017	4.014	1	1.036 (1.001–1.072)	0.045
ASC-12		−0.017	0.031	0.296	1	0.983 (0.926–1.045)	0.586
**Model 2**							
Aura		0.271	0.232	1.368	1	1.311 (0.833–2.064)	0.242
Prophylactic Medication		0.331	0.247	1.801	1	1.392 (0.859–2.258)	0.180
Age at onset of migraine		−0.015	0.012	1.593	1	0.985 (0.963–1.008)	0.207
Duration of migraine		0.022	0.010	4.833	1	1.022 (1.002–1.042)	0.028
Photophobia		1.168	0.476	6.021	1	3.216 (1.265–8.178)	0.014
Phonophobia		−0.080	0.328	0.059	1	0.923 (0.486–1.756)	0.808
Osmophobia		0.898	0.252	12.665	1	2.454 (1.497–4.023)	< 0.001
HIT-6		0.037	0.017	4.693	1	1.038 (1.004–1.073)	0.030
ASC-12		−0.022	0.030	0.543	1	0.978 (0.923–1.037)	0.461

Abbreviations: 95%CI, 95% confidence interval; ASC-12, 12-item Allodynia Symptom Checklist; B, Beta coefficient; BMI, body mass index; df, degrees of freedom; HIT-6, 6-Item Headache Impact Test; OR, odds ratio; SE, standard error; Wald, Wald statistic.

## DISCUSSION

The main finding of the present study was the understanding that migraine patients who perceive food triggers are clinically different from those without food triggers, especially concerning sensory symptoms such as photophobia and osmophobia.

Migraine is characterized by headache accompanied by sensory hypersensitivity of different modalities, such as visual (photophobia), auditory (phonophobia), olfactory (osmophobia), and somatosensory (allodynia), with neurofunctional studies 14 demonstrating that these symptoms may even precede pain. In the present study, patients with photophobia and osmophobia had a higher chance of presenting with food triggers. One hypothesis for this finding could be that, in at least a subset of patients, the perception of food triggers may result from a sensory stimulus, especially osmophobia, since the interpretation of food flavor occurs through the combination of gustatory and olfactory stimuli. This hypothesis is supported by a study 15 describing various odors capable of triggering headaches, with 20.5% of individuals with migraine perceiving the odor of culinary products as a trigger for migraine attacks.

Each year lived with migraine increases the odds of perceiving food triggers by 1.1 time, which may occur due to the greater number of painful experiences over time, but there are studies 16 demonstrating that the time lived with migraine can modify olfactory perception, with a positive correlation between interictal hypersensitivity and disease duration. The same thing occurs with allodynia, the presence and severity of which are associated with disease duration. 17 In the present study, the impact of migraine assessed by the self-administered HIT-6 scale was also associated with a slight increase in the odds of presenting food triggers.

## Limitations

The strength of the present study, which makes it innovative, was the comparison of migraine patients in the presence and absence of food triggers, using a relevant sample and controlling for confounding variables through regression analysis. However, some inherent limitations to its design were observed, thus precluding the inference of causality between the associations. Patients from a single center were included, and the sample was heterogeneous regarding treatment and medication overuse, which may have altered the perception of food triggers. Additionally, the definition of food triggers was based solely on the patient's perception. Therefore, it was not possible to reliably differentiate food triggers from premonitory symptoms of migraine, such as changes in appetite. The validated scales had a considerable amount of missing data and may have failed to show significance due to the smaller valid sample size.

In conclusion, the presence of food triggers was significantly associated with photophobia and osmophobia, suggesting that, at least in some patients, the presence of food triggers may be due to sensory hypersensitivity, especially to osmophobia, since olfactory stimulation is inherent to the act of eating. Osmophobia might be another mechanism by which patients perceive foods as triggers for their migraine attacks. Finally, there is a need for interventions and prospective studies to determine this association.
